# AI in healthcare: navigating opportunities and challenges in digital communication

**DOI:** 10.3389/fdgth.2023.1291132

**Published:** 2023-12-19

**Authors:** George Sun, Yi-Hui Zhou

**Affiliations:** ^1^ Bioinformatics Research Center, North Carolina State University, Raleigh, NC, United States; ^2^ Departments of Biological Sciences and Statistics, North Carolina State University, Raleigh, NC, United States

**Keywords:** remote, AI solutions, telemedicine, transformative healthcare, chatbot, Trust-AI

## Abstract

The landscape of healthcare communication is undergoing a profound transformation in the digital age, and at the heart of this evolution are AI-powered chatbots. This mini-review delves into the role of AI chatbots in digital health, providing a detailed exploration of their applications, benefits, challenges, and future prospects. Our focus is on their versatile applications within healthcare, encompassing health information dissemination, appointment scheduling, medication management, remote patient monitoring, and emotional support services. The review underscores the compelling advantages of AI chatbots. However, it also addresses the significant challenges posed by the integration of AI tools into healthcare communication.

## 1. Introduction

In the contemporary landscape of healthcare, we are witnessing transformative shifts in the way information is disseminated, patient engagement is fostered, and healthcare services are delivered. At the heart of this evolution are AI-powered chatbots, emerging as revolutionary agents of change in healthcare communication. These chatbots, equipped with advanced natural language processing capabilities and machine learning algorithms, hold significant promise in navigating the complexities of digital communication within the healthcare sector.

Healthcare communication is a multifaceted domain that encompasses interactions between patients, healthcare providers, caregivers, and the broader healthcare ecosystem. Effective communication has long been recognized as a fundamental element of quality healthcare delivery. It plays a pivotal role in patient education, adherence to treatment plans, early detection of health issues, and overall patient satisfaction. Nevertheless, the advent of the digital age has presented both opportunities and challenges to traditional healthcare communication approaches.

This mini-review embarks on an exploration of the profound impact that AI-powered chatbots are exerting on healthcare communication, with a particular emphasis on their capacity to catalyze transformative changes in patient behavior and lifestyle choices. Our journey takes us through the evolution of chatbots, from rudimentary text-based systems to sophisticated conversational agents driven by AI technologies. We delve into their multifaceted applications within the healthcare sector, spanning from the dissemination of critical health information to facilitating remote patient monitoring and providing empathetic support services.

The objective of this review is to provide an in-depth examination of the opportunities and challenges presented by AI-powered chatbots in healthcare communication and how they are instrumental in fostering positive shifts in patient behavior and lifestyle choices. Simultaneously, we will address the intricacies, ethical considerations, and potential pitfalls that must be navigated to harness the full potential of this transformative technology in promoting healthier lives and empowering individuals to make informed choices about their well-being.

## 2. Historical evolution of chatbots in healthcare

Initially, chatbots served rudimentary roles, primarily providing informational support and facilitating tasks like appointment scheduling. However, with the relentless advancement of AI and natural language processing (NLP) technologies, chatbots have undergone a metamorphosis, transforming into sophisticated conversational agents with the ability to understand and respond effectively to human language, as evidenced by Khilji et al. (1).

The historical trajectory of chatbots in healthcare reveals pivotal milestones. Notably, the integration of chatbots into healthcare information websites, exemplified by platforms such as WebMD, marked an early stage where chatbots aimed to swiftly address user queries, as elucidated by Goel et al. (2). Subsequent developments saw chatbots seamlessly integrated into electronic health record (EHR) systems, streamlining administrative tasks and enhancing healthcare professional efficiency, as highlighted by Kocakoç (3).

However, the most recent advancements have propelled chatbots into critical roles related to patient engagement and emotional support services. Notably, chatbots like Woebot have emerged as valuable tools in the realm of mental health, engaging users in meaningful conversations and delivering cognitive behavioral therapy (CBT)-based interventions, as demonstrated by Alm and Nkomo (4). This progression underscores the transformative potential of chatbots, including modern iterations like ChatGPT, to transcend their initial role of providing information and actively participate in patient care. As these AI-driven conversational agents continue to evolve, their capacity to positively influence patient behavior and lifestyle choices becomes increasingly evident, reshaping the landscape of healthcare delivery and patient well-being.

## 3. Applications of AI-powered chatbots in healthcare

AI-powered chatbots find diverse applications in the healthcare sector. They have become versatile tools, contributing to various facets of healthcare communication and delivery. One prominent application is health information dissemination. Chatbots embedded in healthcare websites and mobile apps offer users real-time access to medical information, assisting in self-diagnosis and health education (5).

Appointment scheduling and management represent another vital area where chatbots streamline processes. Patients can easily book appointments, receive reminders, and even reschedule appointments through chatbot interactions (6). This convenience not only benefits patients but also reduces the administrative workload on healthcare providers.

In the context of patient engagement, chatbots have emerged as valuable tools for remote monitoring and chronic disease management (7). These chatbots assist patients in tracking vital signs, medication adherence, and symptom reporting, enabling healthcare professionals to intervene proactively when necessary.

### 3.1. AI tools for healthcare providers

AI tools are becoming indispensable in optimizing diagnoses and treatments. Among these tools, AI chatbots stand out as dynamic solutions that offer real-time analytics, revolutionizing healthcare delivery at the bedside. These advancements eliminate unnecessary delays, effectively bridging the gap between diagnosis and treatment initiation.

For instance, DeepMind Health, a pioneering initiative backed by Google, has introduced Streams, a mobile tool infused with AI capabilities, including chatbots. Streams represents a departure from traditional patient management systems, harnessing advanced machine learning algorithms to enable swift evaluation of patient results. This immediacy empowers healthcare providers to promptly identify patients at elevated risk, facilitating timely interventions that can be pivotal in determining patient outcomes.

The trajectory of AI integration in healthcare unmistakably moves towards more streamlined, efficient, and patient-centric modalities, with chatbots at the forefront of this transformation. These AI-driven chatbots serve as virtual assistants to healthcare providers, offering real-time information, decision support, and facilitating seamless communication with patients.

Table 1 presents an overview of current AI tools, including chatbots, employed to support healthcare providers in patient care and monitoring.

**Table 1:** AI Tools Used in Patient Care and Monitoring (incomplete list).

AI tool	Purpose	Year launched
AiCure	Medication adherence	2010
Biofourmis Biovitals	Disease management	2015
CarePredict Tempo	Senior care monitoring	2013
EarlySense	Continuous monitoring for hospitals	2004
IBM Watson Care Manager	Personalized care management	2016
Medtronic Guardian Connect	Glucose monitoring	2018
Philips eCareCoordinator	Chronic disease management	2013
Sense.ly	Virtual nurse assistant	2013
Sensely’s Molly	Virtual health assistant for patient engagement	2013
Vivify Health Platform	Remote patient monitoring	2009
Zephyr Anywhere BioPatch	Vital signs monitoring	2011

### 3.2. AI in telemedicine and remote patient monitoring

In the landscape of digital health, AI-powered chatbots have emerged as transformative tools, reshaping the dynamics of telemedicine and remote patient monitoring. These innovations hold great promise for expanding healthcare access, enhancing patient outcomes, and streamlining healthcare systems. By enabling healthcare services to transcend geographical barriers, chatbots empower patients with unparalleled access to care while relieving the strain on overburdened healthcare facilities (8).

The instrumental role of artificial intelligence becomes evident in the augmentation of telemedicine and remote patient monitoring through chatbot integration. AI-driven chatbots bring personalization, predictive capabilities, and proactive healthcare to the forefront of these digital health strategies.

Within the realm of telemedicine, chatbots equipped with AI capabilities excel at preliminary patient assessments, assisting in case prioritization, and providing valuable decision support for healthcare providers. A noteworthy example is TytoCare’s telehealth platform, where AI-driven chatbots guide patients through self-examination procedures during telemedicine consultations, ensuring the integrity of collected data (9).

In the context of remote patient monitoring, AI-driven chatbots excel at processing and interpreting the wealth of data garnered from wearable devices and smart home systems. Their applications span from predicting exacerbations in chronic conditions such as heart failure and diabetes to aiding in the early detection of infectious diseases like COVID-19 (10, 11). Companies like Biofourmis employ AI chatbots to analyze data from wearable biosensors, remotely monitoring heart failure patients, and preemptively notifying healthcare providers of potential adverse events before they manifest (12). Table 2 provides an overview of popular AI-powered Telehealth chatbot tools and their annual revenue.

**Table 2:** List of AI tools in telemedicine and remote patient monitoring.

AI tool	Year launched	Estimated annual revenue
Ada health	2016	$133.7M
Biofourmis	2015	$151.7M
Kareo	2022	$70M
Teladoc	2022	$2.4B
TytoCare	2012	$21M

## 4. Challenges and limitations

While AI-powered chatbots have been instrumental in transforming the healthcare landscape, their implementation and integration have many challenges. This section outlines the major limitations and hurdles in the deployment of AI chatbot solutions in healthcare.

### 4.1. Data privacy and security

Federated learning is an emerging research topic that addresses the challenges of preserving data privacy and security in the context of machine learning, including AI chatbots. It allows multiple participants to collaboratively train a machine learning model without sharing their raw data. Instead, the model is trained locally on each participant’s device or server using their respective data, and only the updated model parameters are shared with a central server or coordinator.

The concept of federated learning has gained attention due to its potential to overcome privacy concerns associated with sharing sensitive healthcare data (13). With federated learning, healthcare organizations, researchers, and other stakeholders can collectively develop robust machine learning models while keeping patient data decentralized and secure.

One notable algorithm in the field of federated learning is the Hybrid Federated Dual Coordinate Ascent (HyFDCA), proposed in 2022 (14). HyFDCA focuses on solving convex optimization problems within the hybrid federated learning setting. It employs a primal-dual setting, where privacy measures are implemented to ensure the confidentiality of client data. By using HyFDCA, participants in federated learning settings can collaboratively optimize a common objective function while protecting the privacy and security of their local data. This algorithm introduces privacy steps to guarantee that client data remains private and confidential throughout the federated learning process.

As federated learning continues to evolve, researchers and practitioners are actively exploring various techniques and algorithms to address the complexities of healthcare data privacy, security, and regulatory compliance (15). These efforts aim to strike a balance between leveraging the power of AI chatbots for improved healthcare outcomes while safeguarding the privacy and confidentiality of sensitive patient information.

### 4.2. Algorithm bias and fairness

As AI chatbots increasingly permeate healthcare, they bring to light critical concerns about algorithmic bias and fairness (16). AI, particularly Machine Learning, fundamentally learns patterns from the data they are trained on Goodfellow et al. (17). If the training data lacks diversity or contains inherent bias, the resultant chatbot models may mirror these biases (18). Such a scenario can potentially amplify healthcare disparities, as it may lead to certain demographics being underserved or wrongly diagnosed (19).

Given the potential for adverse outcomes, it becomes imperative to ensure that the development and deployment of AI chatbot models in healthcare adhere to principles of fairness and equity (16). Achieving this can promote equitable healthcare access and outcomes for all population groups, regardless of their demographic characteristics (20).

Nonetheless, the problem of algorithmic bias is not solely restricted to the nature of the training data. Several other factors can introduce and perpetuate bias in AI chatbot models. One of these is biased feature selection, where selecting features used to train the model can lead to biased outcomes, particularly if these features correlate with sensitive attributes such as race or gender (21).

Another factor is the distribution of classes in the training data. If certain classes are overrepresented or underrepresented, the resultant chatbot model may be skewed towards predicting the overrepresented classes, thereby leading to unfair outcomes for the underrepresented classes (22).

Moreover, model overfitting, where a model learns the training data too well and is unable to generalize to unseen data, can also exacerbate bias (21). This is particularly concerning in healthcare, where the chatbot’s predictions may influence critical decisions such as diagnosis or treatment (23).

Consequently, addressing the issue of bias and ensuring fairness in healthcare AI chatbots necessitates a comprehensive approach. This includes being cognizant of the potential for bias in the data and the model development process, as well as actively implementing strategies to mitigate such bias (24). Furthermore, ongoing monitoring of deployed chatbot models is also required to detect and correct any emergent bias. Only through such multi-faceted efforts can we hope to leverage the potential of AI chatbots in healthcare while ensuring that their benefits are equitably distributed (16).

### 4.3. Explainability and trust

In the realm of AI-driven communication, a fundamental challenge revolves around elucidating the models’ decision-making processes, a challenge often denoted as the “black box” problem (25). The complex nature of these systems frequently shrouds the rationale behind their decisions, presenting a substantial barrier to cultivating trust in their application.

The AI community has made commendable progress in confronting these challenges. Explainable AI (XAI) emerges as a pivotal approach to unravel the intricacies of AI models, enhancing not only their performance but also furnishing users with insights into the reasoning behind their outputs (26). Techniques such as LIME (Local Interpretable Model-agnostic Explanations) (27) and SHAP (SHapley Additive exPlanations) (28) have played a crucial role in illuminating the decision-making processes, thereby rendering the “black box” more interpretable.

Trust AI assumes a critical role in navigating complexities, particularly in AI-powered chatbots. Serving as a link between theoretical analytical expressions and the numerical models derived through Machine Learning, Trust AI addresses the challenge of explainability. The nuanced nature of human-machine interactions demands a delicate balance between analytical rigor and user-friendly outcomes. A recent study adopted a user’s mental model (29). However, there is still a long way to go in enhancing Trust AI. We need the multifaceted Trust AI approach to augment transparency and interpretability, fostering trust in AI-driven communication systems.

The challenge of explainability in AI-powered communication intertwines with establishing trust, amplified in dynamic chatbot interactions. Advances in XAI methodologies, ethical frameworks, and interpretable models represent indispensable strides in demystifying the “black box” within chatbot systems. Ongoing efforts are paramount to instill confidence in AI-driven communication, especially involving chatbots.

### 4.4. Regulatory approval and standardization

Navigating regulatory landscapes can present significant hurdles for AI chatbots in healthcare (30). Regulatory bodies like the Food and Drug Administration (FDA) in the US or the European Medicines Agency (EMA) in Europe have rigorous processes for granting approval to AI chatbot-based medical devices and solutions. These processes, while critical for ensuring safety and efficacy, can be time-consuming and resource-intensive.

Moreover, the rapidly evolving nature of AI chatbot technology and the lack of standardization in AI chatbot applications further complicate the process of regulatory assessment and oversight (31). While efforts are underway to adapt regulatory frameworks to the unique challenges posed by AI chatbots, this remains an area of ongoing complexity and challenge.

## 5. Conclusion

In conclusion, while AI chatbots hold immense potential to transform healthcare by improving access, patient care, and efficiency, they face significant challenges related to data privacy, bias, interoperability, explainability, and regulation. Addressing these challenges through technological advancements, ethical considerations, and regulatory adaptation is crucial for unlocking the full potential of AI chatbots in revolutionizing healthcare delivery and ensuring equitable access and outcomes for all.
